# Association between epidermal growth factor gene +61A/G polymorphism and the risk of hepatocellular carcinoma: a meta-analysis based on 16 studies

**DOI:** 10.1186/s12885-015-1318-6

**Published:** 2015-04-25

**Authors:** Guoping Jiang, Ke Yu, Lifang Shao, Xiaobo Yu, Chen Hu, Pei Qian, Haiyang Xie, Jinjun Li, Jie Zheng, Shusen Zheng

**Affiliations:** 1 Collaborative Innovation Center for Diagnosis and Treatment of Infectious Diseases, The First Affiliated Hospital, School of Medicine, Zhejiang University, 79 Qingchun Rd, Hangzhou, 310003 China; 2 Key Laboratory of Combined Multi-organ Transplantation of Ministry of Public Health, Hangzhou, China; 3 Zhejiang-California International Nanosystems Institute, Zhejiang University, Hangzhou, Zhejiang 310029 China; 4 State Key Laboratory of Oncogenes and Related Genes, Shanghai Cancer Institute, Shanghai, China

**Keywords:** Hepatocellular carcinoma, Epidermal growth factor, Polymorphism, Susceptibility, Meta-analysis

## Abstract

**Background:**

The association between epidermal growth factor (EGF) gene +61A/G polymorphism (rs4444903) and hepatocellular carcinoma (HCC) susceptibility has been widely reported, but the results were inconsistent. To clarify the effect of this polymorphism on HCC risk, a meta-analysis was performed.

**Methods:**

The PubMed, Embase, Cochrane Library, Web of Science, Chinese BioMedical Literature (CBM), Wanfang and Chinese National Knowledge Infrastructure (CNKI) databases were systematically searched to identify relevant studies published up to December 2013. Data were extracted independently by two authors. Odds ratios (ORs) and 95% confidence intervals (95% CIs) were calculated to assess the strength of association.

**Results:**

A total of 16 studies including 2475 HCC cases and 5381 controls were included in this meta-analysis. Overall, a significantly increased HCC risk was observed under all genetic models (G vs. A: OR = 1.383, *P* < 0.001, 95% CI: 1.174-1.629; GG vs. GA + AA: OR = 1.484, *P* < 0.001, 95% CI: 1.198-1.838; GG + GA vs. AA: OR = 1.530, *P* < 0.001, 95% CI: 1.217-1.924; GG vs. AA: OR = 1.958, *P* < 0.001, 95% CI: 1.433-2.675; GA vs. AA: OR = 1.215, *P* = 0.013, 95% CI: 1.041-1.418). In the subgroup analyses by ethnicity, a significant association with HCC risk was found in Asian populations (G vs. A: OR = 1.151, *P* = 0.001, 95% CI: 1.056-1.255), European populations (G vs. A: OR = 1.594, *P* = 0.027, 95% CI: 1.053-2.413, and African populations (G vs. A: OR = 3.599, *P* < 0.001, 95% CI: 2.550-5.080), respectively.

**Conclusions:**

Our study shows that EGF +61A/G polymorphism is significantly associated with the increased HCC risk, especially in Asian populations. Further large-scale and well-designed studies are required to confirm this conclusion.

**Electronic supplementary material:**

The online version of this article (doi:10.1186/s12885-015-1318-6) contains supplementary material, which is available to authorized users.

## Background

Hepatocellular carcinoma (HCC) is the fifth most common cancer and the third leading cause of cancer-related death worldwide [1]. The estimated annual number of cases exceeds 500 000, with a mean annual incidence of around 3-4% [2]. Most cases of HCC (about 80%) occur in eastern Asia and sub-Saharan Africa, and China alone accounts for more than 50% of the total cases [3]. Despite advances in the diagnosis and treatment of HCC, it still has poor prognosis with a five-year survival rate of 5% in developing countries [4]. Carcinogenesis of HCC is a complex, multistep and multifactorial process. Major risk factors for development of HCC are chronic infection with hepatitis B virus (HBV) or hepatitis C virus (HCV), liver cirrhosis, habitual alcohol abuse, high cigarette smoking, and exposure to aflatoxin B1 [3,5]. However, not all individuals with exposure to the risk factors develop HCC. Therefore, other causes, including genetic factors, might play important roles in the pathogenesis of HCC.

Epidermal growth factor (EGF) was first isolated in 1962 [6]. It stimulates proliferation, differentiation and tumorigenesis of epidermal and epithelial tissues by binding to its receptor (EGFR) and, hence, activating several signal pathways [7,8]. EGF is a mitogen for adult and fetal hepatocytes grown in culture, and its expression is up-regulated during liver regeneration [9]. Mounting evidence supports a role for EGF in malignant transformation, tumor growth and progression [10]. The EGF gene is located on chromosome 4q25-27 and contains 24 exons and 23 introns. The EGF +61A/G polymorphism (rs4444903) is a common single nucleotide polymorphism (SNP) in the 5′-untranslated region (5′-UTR) of the EGF gene, modulating the transcription of EGF gene and hence affecting serum levels of EGF [11]. For now, there are a number of studies conducted to examine the association between EGF +61A/G polymorphism and HCC susceptibility, but the results remain controversial and inconclusive [12-16]. These disparate findings may be due partly to insufficient power, false-positive results and ethnic diversity.

Meta-analysis offers a powerful means of overcoming the problems associated with small sample sizes, and particularly, of overcoming the inadequate statistical powers of genetic studies on complex traits [17]. Therefore, in this study, we performed a meta-analysis from all eligible studies to clarify the relationship between EGF +61A/G polymorphism and HCC risk.

## Methods

This meta-analysis followed the Preferred Reporting Items for Systematic Reviews and Meta-analyses (PRISMA) criteria [18].

### Literature searching strategy

We conducted a computerized literature search of PubMed, Embase, Cochrane Library, Web of Science, Chinese BioMedical Literature (CBM), Wanfang and Chinese National Knowledge Infrastructure (CNKI) databases to identify all potential studies published up to December 31, 2013. The following keywords and subject terms were included in searching: “EFG” or “Epidermal growth factor”, “liver cancer” or “hepatocellular carcinoma” or “HCC”, and “polymorphism” or “variant” or “allele”. References of retrieved articles and review articles were also screened.

### Inclusion criteria

Studies included in the meta-analysis had to meet all the following criteria: (1) evaluating the association between EGF +61A/G polymorphism and HCC risk, (2) using unrelated individuals, (3) providing sufficient data for estimating an odds ratio (OR) with its 95% confidence interval (CI), (4) using case–control, cohort or cross-sectional design, (5) published in English or Chinese. The corresponding authors were contacted to obtain missing information, and some studies were excluded if critical missing information was not obtained. Reviews, case reports, family-based studies, case-only studies, and studies without sufficient data were all excluded. When a study reported results on different subpopulations based on ethnicity or geographical region, we treated each subpopulation as a separate comparison. If more than one article was published using the same subjects, only the study with the largest sample size was selected.

### Data extraction

All data were extracted independently by two investigators (Lifang Shao and Xiaobo Yu). Disagreement was resolved by discussion. The following data were extracted: authors, name of journal, year of publication, ethnicity and country of study population, inclusion and exclusion criteria, characteristics of cases and controls, numbers of HCC cases and controls, matching criteria, source of controls, HCC confirmation, study design, genotyping methods, genotype frequencies of cases and controls, and interactions between environment factors or genes.

### Quality score assessment

Quality of studies was independently assessed by the same two investigators (Lifang Shao and Xiaobo Yu) according to a set of predetermined criteria (Additional file 1: Table S1), which was extracted and modified from previous studies [19,20]. These scores were based on traditional epidemiological considerations, as well as cancer genetic issues. Any disagreement was resolved by discussion between the two investigators. The total scores ranged from 0 (worst) to 24 (best). Studies scoring <16 were classified as “low quality”, and those scoring ≥16 as “high quality”.

### Statistical analysis

The unadjusted OR with 95% CI was used to assess the strength of the association between EGF +61A/G polymorphism and HCC risk. The pooled ORs were performed under the allelic contrast (G versus A), co-dominant model (homozygote comparison: GG versus AA, heterozygote comparison: GA versus AA), dominant model (GG + GA versus AA), and recessive model (GG versus GA + AA), respectively. Between-study heterogeneity was measured using a *Q*-statistic test [21] and an *I*-square statistic [22]. *P* less than 0.10 (*P* < 0.10) was considered representative of significant statistical heterogeneity because of the low power of the statistic. *I*^*2*^ ranges between 0 and 100%, and represents the proportion of between-study variability that can be attributed to heterogeneity rather than chance. *I*^*2*^ values of 25%, 50%, and 75% were defined as low, moderate, and high estimates. If the significant Q-statistic indicated heterogeneity across studies, the random-effects model (DerSimonian and Laird method) was used, otherwise the fixed-effects model (Mantel-Haenszel method) was adopted [23]. The *Z* test was used to assess the significance of the pooled OR and a *P*-value less than 0.05 (*P* < 0.05) was considered significant.

Subgroup analyses were stratified by racial descent, study quality, source of controls, type of controls, and number of cases, respectively. Furthermore, meta-regression analysis [24] was performed to investigate five potential sources of heterogeneity including ethnicity (Asian populations versus not Asian populations), study quality (high quality studies versus low quality studies), source of controls (Hospital-based versus Population-based), type of controls (healthy controls versus controls with chronic liver diseases) and number of cases (<100 versus ≥100). Statistical significance was defined as a *P*-value less than 0.10 (*P* < 0.10) because of the relatively weak statistical power.

To evaluate the stability of the results, sensitivity analyses were performed by sequential omission of individual studies under various comparisons in overall and Asian populations, respectively. Publication bias was investigated by funnel plot. Funnel plot asymmetry was assessed by the method of Egger’s linear regression test [25]. Hardy-Weinberg equilibrium (HWE) was tested by the *χ*^*2*^ test. All *P*-values were two-sided. Data analyses were performed using the software Stata version 11.0 (StataCorp LP, College Station, TX, USA).

## Results

### Eligible studies

**Figure 1:**
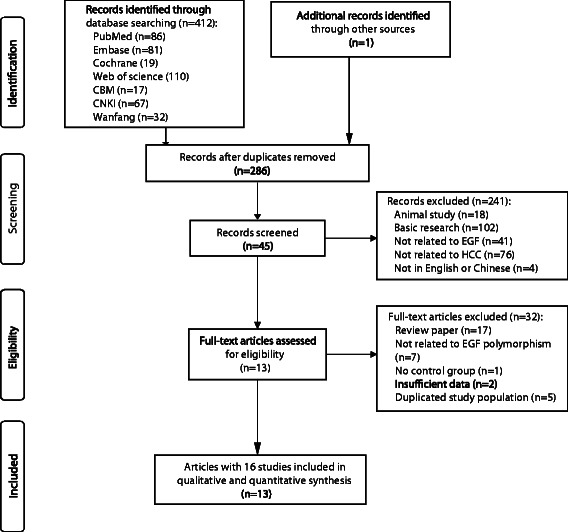
Flow diagram of the study selection process.

The present study met the PRISMA statement (Additional file 2: Checklist S1). A total of 413 potentially relevant records were initially obtained through searching the databases. After removing 127 duplications, 241 records were excluded because of obvious irrelevance to our study aim by browsing the titles and abstracts. According to the inclusion criteria, 32 of the remaining 45 records were further excluded by review of the full texts. The flow chart of the selection process was shown in Figure 1. In total, 13 articles were eligible, of which three provided the data in different populations [12,13,26]. We treated each population as a separate study. As a result, 16 studies (13 articles) including 2475 HCC cases and 5381 controls were identified and included in this meta-analysis [12-16,26-33].

### Characteristics of studies and subjects

The main characteristics of the 16 included studies were listed in Table 1. All articles were published in English except for one [26]. Of all the eligible studies, 9 were conducted in Asian populations, 2 in European populations, 2 in African populations, and 3 in mixed populations with more than 72% Caucasians. In all the studies, the cases were histologically confirmed (11 studies) or diagnosed by elevated α-fetoprotein and distinct iconography changes (abdominal ultrasound and Triphasic computed tomography). All the controls were free of cancer. Two studies used healthy populations, 4 studies used patients with chronic liver diseases (HBV infection, HCV infection, cirrhosis), and 10 studies included both healthy subjects and patients with chronic liver diseases as controls. Seven studies matched in age, 10 studies matched in gender, and 9 studies matched in hepatitis virus infection status. The sample size of the total participants ranged from 80 to 1774, with a mean of 491. The quality scores for the individual studies ranged from 11.5 to 21, with 9 out of the 16 studies classified as high quality. Fifteen studies used peripheral blood, and one study used either blood or liver tissue to extract genome DNA. Thirteen studies used the polymerase chain reaction-restriction fragment length polymorphism assay (PCR-RFLP), and three studies used Taqman method to genotype the EGF +61 A/G polymorphism. While genotyping, 10 studies repeated a portion of samples, and only 4 studies described use of blindness of the status of DNA samples. The genotype distribution in the controls of all studies was consistent with HWE.

**Table 1:** **Main characteristics of eligible studies included in the meta-analysis**

First author (year)	Country (Ethnicity)	Source of controls	Type of controls	Sample origin	Genotyping methods	Sample size (case/control)	Genotype frequency (case/control)			G allele frequency	HWE (Y/N)	Quality score
							GG	GA	AA			
Tanabe-FRA (2008) [12]	France (European)	HB	Cirrhosis	Peripheral blood	PCR-RFLP	44/77	15/12	17/37	12/28	39.6%	Y	13.5
Tanabe-USA (2008) [12]	USA (mixed)	HB	HBV/HCV/Cirrhosis	Peripheral blood	PCR-RFLP	59/148	23/32	27/65	9/51	43.6%	Y	14.5
Qi (2009) [16]	China (Asian)	HB and PB	Healthy/HBV	Peripheral blood	PCR-RFLP	215/380	102/182	98/160	15/38	68.9%	Y	21
Wang-GX (2009) [26]	China (Asian)	HB	Healthy/HBV	Peripheral blood	PCR-RFLP	376/477	190/208	154/221	32/48	66.8%	Y	17.5
Wang-JS (2009) [26]	China (Asian)	HB	Healthy/HBV	Peripheral blood	PCR-RFLP	186/198	107/93	65/88	14/17	69.2%	Y	18
Li (2010) [31]	China (Asian)	HB and PB	Healthy/Cirrhosis	Peripheral blood	PCR-RFLP	186/338	96/161	82/145	8/32	69.1%	Y	19.5
Abu Dayyeh (2011) [29]	USA (mixed)	HB	HCV	Peripheral blood	PCR-RFLP	66/750	26/178	25/350	15/222	47.1%	Y	16.5
Chen (2011) [30]	China (Asian)	HB	Healthy/HBV/Cirrhosis	Peripheral blood	PCR-RFLP	120/240	62/106	51/110	7/24	67.1%	Y	19
Abbas (2012) [27]	Egypt (African)	HB	Healthy/HCV/Cirrhosis	Peripheral blood	PCR-RFLP	20/60	7/9	9/28	4/23	38.3%	Y	12
Cmet (2012) [33]	Italy (European)	HB and PB	Healthy/HBV	Peripheral blood	PCR-RFLP	18/361	4/66	10/172	4/123	42.1%	Y	16
Shi (2012) [28]	China (Asian)	HB	Healthy	Peripheral blood	PCR-RFLP	73/117	18/13	31/52	24/52	33.3%	Y	13.5
El-Bendary (2013) [32]	Egypt (African)	HB	HCV/Cirrhosis	Peripheral blood	PCR-RFLP	133/105	57/9	43/36	33/60	25.7%	Y	12
Suenaga (2013) [14]	Japan (Asian)	HB	Healthy/HBV/HCV	Peripheral blood or liver tissue	PCR-RFLP	208/290	108/161	89/104	11/25	73.4%	Y	11.5
Wu (2013) [15]	China (Asian)	HB and PB	Healthy/HBV	Peripheral blood	TaqMan	404/1370	206/647	153/576	45/147	68.2%	Y	17.5
Yuan-USA (2013) [13]	USA (mixed)	PB	Healthy	Peripheral blood	TaqMan	117/225	28/63	61/102	28/60	50.7%	Y	19
Yuan-CHN (2013) [13]	China (Asian)	HB	Healthy/HBV/HCV	Peripheral blood	TaqMan	250/245	25/20	99/107	126/118	30.0%	Y	15

HB, Hospital-based; PB, Population-based; HBV, control subjects were hepatitis B virus carriers; HCV, control subjects were hepatitis C virus carriers; PCR-RFLP, polymerase chain reaction-restriction fragment length polymorphism; HWE: Hardy-Weinberg equilibrium in control population; Y, yes; N, no.

### Meta-analysis results

**Figure 2:**
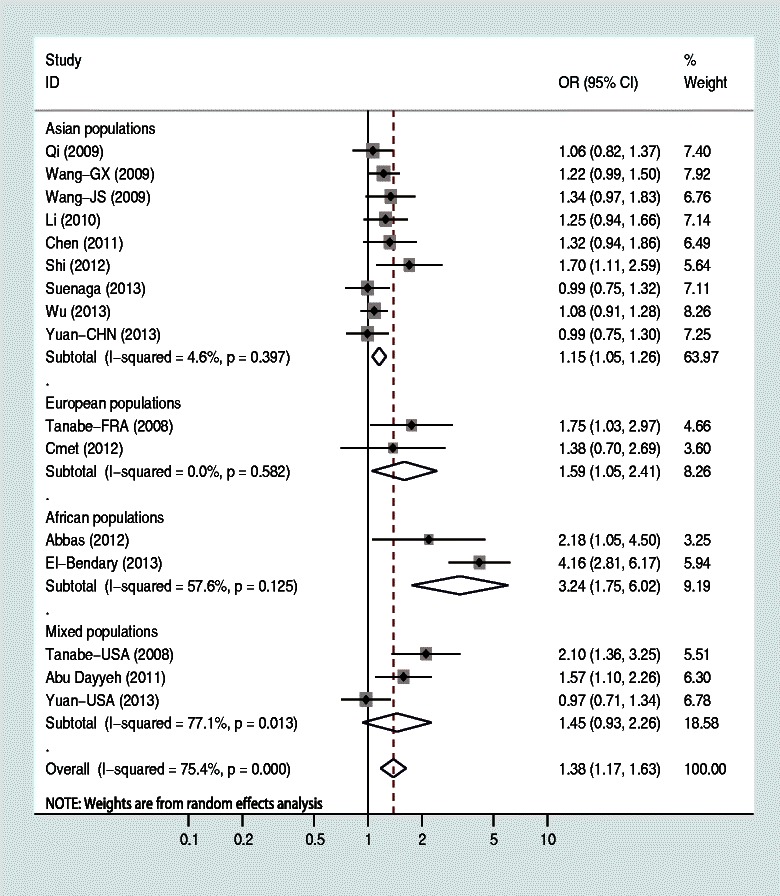
Forest plot for the association between *EGF* +61A/G polymorphism and HCC risk stratified according to different ethnicities (G vs. A). For each study, the estimate of OR and its 95% CI is plotted with a diamond (◆) and a horizontal line. The size of a box (gray square) is proportional to the weight that the study has in calculating the summary effect estimate (`). The center of the diamond indicates the OR and the ends of the diamond correspond to the 95% CI.

The frequency of +61G allele was 65% in Asian controls, 42% in European controls, and 30% in African controls. There were significant differences in terms of +61G allele frequency among the three major ethnicities (P < 0.001). Table 2 indicated the associations between EGF +61A/G polymorphism and HCC risk. Overall, the results of pooling all studies showed that the EGF +61A/G polymorphism was significantly associated with an increased HCC risk under all genetic models (G vs. A: OR = 1.383, *P* < 0.001, 95% CI: 1.174-1.629, *I*^*2*^ = 75.4%, *P*_heterogeneity_ < 0.001, Figure 2; GG vs. GA + AA: OR = 1.484, *P* < 0.001, 95% CI: 1.198-1.838, *I*^*2*^ = 69.3%, *P*_heterogeneity_ < 0.001; GG + GA vs. AA: OR = 1.530, *P* < 0.001, 95% CI: 1.217-1.924, *I*^*2*^ = 53.5%, *P*_heterogeneity_ = 0.006; GG vs. AA: OR = 1.958, *P* < 0.001, 95% CI: 1.433-2.675, *I*^*2*^ = 65.2%, *P*_heterogeneity_ < 0.001; GA vs. AA: OR = 1.215, *P* = 0.013, 95% CI: 1.041-1.418, *I*^*2*^ = 19.7%, *P*_heterogeneity_ = 0.229) (Table 2).

**Table 2:** **Main results of meta-analysis for EGF +61A/G polymorphism and HCC risk**

Subgroup	No.comparisons	Sample size (case/control)	GG vs. GA + AA		GG + GA vs. AA		GG vs. AA		GA vs. AA		\G vs. A	
			OR (95% CI)	*I*^*2*^(%)	OR (95% CI)	*I*^*2*^(%)	OR (95% CI)	*I*^*2*^(%)	OR (95% CI)	*I*^*2*^(%)	OR (95% CI)	*I*^*2*^(%)
Overall	16	2475/5381	1.48 (1.20-1.84)*	69.3**	1.53 (1.22-1.92)*	53.5**	1.96 (1.43-2.68)*	65.2**	1.22 (1.04-1.42)*	19.7	1.38 (1.17-1.63)*	75.4**
Racial descent												
Asian	9	2018/3655	1.19 (1.06-1.34)*	24.9	1.20 (1.01-1.43)*	16.3	1.40 (1.14-1.71)*	1.3	1.10 (0.92-1.33)	23.5	1.15 (1.05-1.26)*	4.6
European	2	62/438	2.10 (1.07-4.13)*	12.6	1.62 (0.84-3.13)	0.0	2.51 (1.11-5.71)*	0.0	1.30 (0.64-2.63)	0.0	1.59 (1.05-2.41)*	0.0
African	2	153/165	6.33 (3.39-11.83)*	46.8	3.69 (2.23-6.10)*	0.0	9.34 (4.61-18.91)*	19.4	2.11 (1.21-3.67)*	0.0	3.60 (2.55-5.08)*	57.6
Mixed	3	242/1123	1.55 (0.79-3.06)	77.3**	1.52 (1.08-2.16)*	46.9	1.94 (0.87-4.33)	73.1**	1.37 (0.94-2.00)	11.0	1.45 (0.93-2.26)	77.1**
Study quality												
High quality	9	1688/4339	1.22 (1.08-1.38)*	21.0	1.26 (1.04-1.52)*	0.0	1.36 (1.11-1.67)*	0.0	1.16 (0.94-1.42)	0.0	1.18 (1.08-1.29)*	0.0
Asian	6	1487/3003	1.22 (1.07-1.38)*	0.0	1.23 (0.99-1.54)	8.5	1.34 (1.06-1.69)*	0.0	1.13 (0.89-1.43)	23.2	1.17 (1.06-1.29)*	0.0
Others	3	201/1336	1.29 (0.65-2.58)	69.0**	1.32 (0.92-1.91)	0.0	1.45 (0.94-2.23)	37.6	1.25 (0.84-1.85)	0.0	1.22 (0.98-1.52)	50.0
Low quality	7	787/1042	2.28 (1.23-4.23)*	82.7**	1.90 (1.17-3.09)*	74.3**	3.06 (1.62-5.76)*	72.9**	1.45 (1.02-2.07)*	44.1**	1.74 (1.14-2.65)*	87.1**
Asian	3	531/652	1.31 (0.72-2.38)	69.6**	1.14 (0.86-1.51)	49.3	1.59 (1.05-2.43)*	32.8	1.07 (0.80-1.44)	48.8	1.14 (0.85-1.53)	60.5**
Others	4	256/390	3.57 (1.94-6.60)*	53.4**	2.89 (1.98-4.20)*	22.6	5.67 (3.52-9.14)*	41.6	1.88 (1.25-2.82)*	0.0	2.47 (1.61-3.80)*	65.8**
Source of controls												
Population-based	5	940/1451	1.04 (0.88-1.24)	0.0	1.28 (0.99-1.67)	0.0	1.26 (0.94-1.67)	0.0	1.29 (0.98-1.71)	0.0	1.09 (0.96-1.24)	0.0
Asian	3	805/1017	1.07 (0.89-1.29)	0.0	1.29 (0.94-1.77)	9.1	1.32 (0.95-1.84)	0.0	1.26 (0.90-1.76)	40.1	1.10 (0.95-1.27)	0.0
Others	2	135/434	0.87 (0.54-1.40)	0.0	1.27 (0.79-2.02)	0.0	1.07 (0.60-1.90)	0.0	1.37 (0.83-2.25)	0.0	1.04 (0.78-1.39)	0.0
Hospital-based	15	2358/3930	1.59 (1.27-1.99)*	67.3**	1.57 (1.21-2.04)*	58.5**	2.10 (1.49-2.96)*	66.6**	1.18 (1.00-1.39)	27.6	1.44 (1.21-1.72)*	75.8**
Asian	9	2018/2638	1.23 (1.09-1.39)*	20.2	1.16 (0.97-1.40)	26.7	1.38 (1.11-1.71)*	22.6	1.06 (0.88-1.28)	24.6	1.16 (1.06-1.27)*	17.6
Others	6	340/1292	2.79 (1.72-4.53)*	54.0**	2.31 (1.70-3.14)*	37.9	3.77 (2.07-6.88)*	55.5**	1.61 (1.16-2.25)*	0.0	2.08 (1.44-2.98)*	69.0**
Type of controls												
Healthy controls	8	1153/1708	1.10 (0.94-1.29)	34.3	1.40 (1.11-1.76)*	7.8	1.44 (1.11-1.86)*	31.7	1.35 (1.05-1.72)*	0.0	1.18 (1.00-1.41)	43.4**
Asian	5	998/1254	1.11 (0.94-1.32)	36.8	1.36 (1.04-1.79)*	0.0	1.47 (1.09-1.97)*	8.5	1.28 (0.96-1.71)	0.0	1.14 (1.00-1.30)*	4.2
Others	3	155/454	1.01 (0.65-1.57)	51.2	1.95 (0.81-4.73)	58.4**	2.08 (0.59-7.31)	65.1**	1.54 (0.96-2.47)	16.6	1.59 (0.77-3.29)	75.5**
Patients with chronic liver diseases	12	1395/2696	1.74 (1.29-2.36)*	70.0**	1.87 (1.30-2.67)*	58.7**	2.58 (1.60-4.16)*	69.9**	1.36 (1.09-1.70)*	36.0	1.56 (1.23-1.99)*	76.9**
Asian	5	1055/1424	1.19 (1.01-1.40)*	11.0	1.64 (0.95-2.84)	61.7**	1.77 (1.02-3.06)*	59.1**	1.53 (0.86-2.72)	62.6**	1.17 (1.03-1.32)*	23.3
Others	7	340/1272	2.73 (1.74-4.28)*	45.2**	2.22 (1.64-3.01)*	34.2	3.53 (2.01-6.20)*	47.5**	1.55 (1.12-2.16)*	0.0	1.98 (1.41-2.79)*	64.4**
Number of cases												
≥100	10	2195/3868	1.27 (1.01-1.60)*	72.6**	1.44 (1.07-1.94)*	66.1**	1.66 (1.13-2.43)*	71.8**	1.24 (0.98-1.58)	39.7**	1.26 (1.03-1.53)*	80.7**
Asian	8	1945/3538	1.17 (1.04-1.32)*	0.0	1.16 (0.97-1.40)	16.6	1.34 (1.08-1.65)*	0.0	1.09 (0.90-1.32)	31.3	1.13 (1.04-1.24)*	0.0
Others	2	250/330	2.50 (0.26-23.99)	95.8**	2.15 (0.63-7.34)	90.5**	3.27 (0.28-37.81)	95.5**	1.62 (1.08-2.44)*	36.5	2.00 (0.48-8.32)	96.9**
<100	6	280/1513	2.28 (1.68-3.10)*	0.0	1.79 (1.31-2.45)*	0.0	2.82 (1.93-4.13)*	0.0	1.40 (1.00-1.96)	0.0	1.74 (1.43-2.12)*	0.0
Asian	1	73/117	2.62 (1.20-5.74)*	-	1.63 (0.89-3.00)	-	3.00 (1.27-7.10)*	-	1.29 (0.67-2.49)	-	1.70 (1.11-2.59)*	-
Others	5	207/1396	2.22 (1.59-3.10)*	0.0	1.85 (1.28-2.66)*	0.0	2.78 (1.82-4.25)*	0.0	1.44 (0.97-2.14)	0.0	1.75 (1.41-2.18)*	0.0

OR, odds ratio; 95% CI, 95% confidence interval.

*Significant results, P-value <0.05.

**Random effect estimate.

In the subgroup analyses based upon ethnicity, a significantly elevated association between EGF +61A/G polymorphism and HCC risk was observed in Asian populations (G vs. A: OR = 1.151, *P* = 0.001, 95% CI: 1.056-1.255, *I*^*2*^ = 4.6%, *P*_heterogeneity_ = 0.397), European populations (G vs. A: OR = 1.594, *P* = 0.027, 95% CI: 1.053-2.413, *I*^*2*^ = 0.0%, *P*_heterogeneity_ = 0.582), and African populations (G vs. A: OR = 3.599, *P* < 0.001, 95% CI: 2.550-5.080, *I*^*2*^ = 57.6%, *P*_heterogeneity_ = 0.125), respectively (Figure 2). When stratifying by study quality, the results showed that EGF +61A/G polymorphism was associated with an increased HCC risk both in high-quality studies (G vs. A: OR = 1.178, *P* < 0.001, 95% CI: 1.077-1.289, *I*^*2*^ = 0.0%, *P*_heterogeneity_ = 0.539) and in low-quality studies (G vs. A: OR = 1.740, *P* = 0.010, 95% CI: 1.144-2.648, *I*^*2*^ = 87.1%, *P*_heterogeneity_ < 0.001). In the subgroup analyses by source of controls, the results showed that EGF +61A/G polymorphism was significantly associated with HCC risk in hospital-based studies (G vs. A: OR = 1.439, *P* < 0.001, 95% CI: 1.205-1.719, *I*^*2*^ = 75.8%, *P*_heterogeneity_ < 0.001), but not in population-based studies (G vs. A: OR = 1.087, *P* = 0.202, 95% CI: 0.956-1.236, *I*^*2*^ = 0.0%, *P*_heterogeneity_ = 0.815). Furthermore, according to chronic liver disease status in Asian controls, a significant association between EGF +61A/G polymorphism and HCC risk was obtained in patients with chronic liver diseases (G vs. A: OR = 1.165, *P* = 0.017, 95% CI: 1.028-1.321, *I*^*2*^ = 23.3%, *P*_heterogeneity_ = 0.266), and in healthy controls (G vs. A: OR = 1.142, *P* = 0.043, 95% CI: 1.004-1.299, *I*^*2*^ = 4.2%, *P*_heterogeneity_ = 0.383) (Table 2).

### Heterogeneity analysis

Q-statistic indicated statistically significant heterogeneity among all studies under all genetic models except for heterozygote comparison (Table 2). However, in the subgroup analyses by ethnicity, the between-study heterogeneity was not observed in Asian populations, European populations or African populations. Moreover, meta-regression indicated that both ethnicity and study quality significantly contributed to the heterogeneity for EGF +61A/G polymorphism (Table 3).

**Table 3:** **Main results of meta-regression for EGF +61A/G polymorphism and HCC risk**

Factor	GG vs. GA + AA		GG + GA vs. AA		GG vs. AA		GA vs. AA		G vs. A	
	*t*	*P*	*t*	*P*	*t*	*P*	*t*	*P*	*t*	*P*
Ethnicity	2.18	0.047	1.93	0.075	2.05	0.060	1.54	0.146	2.57	0.022
Study quality	2.06	0.059	1.35	0.199	2.36	0.034	0.79	0.443	1.83	0.088

### Sensitivity analysis and publication bias

**Figure 3:**
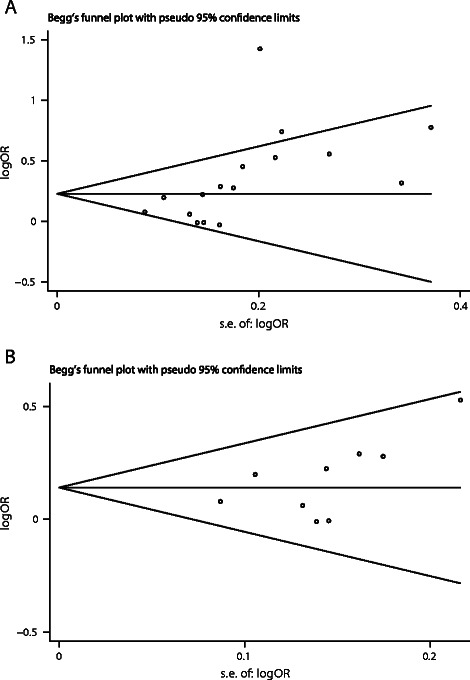
Begg’s funnel plot of the Egger’s test for publication bias of EGF +61A/G polymorphism and HCC risk (G vs. A). **A**: Overall populations; **B**: Asian populations. The horizontal line in the funnel plot indicates summary estimate, whereas the sloping lines indicate the expected 95% confidence intervals for a given standard error.

Sensitivity analysis was performed by sequential omission of individual studies. The pooled ORs were consistently significant in overall populations or Asian populations by omitting one study at a time under the allelic contrast, recessive model and homozygote comparison, suggesting robustness of our results. Funnel plots and Egger’s test were performed to assess publication bias. The results showed that bias may exist in overall populations (G vs. A: t = 2.62, *P* = 0.020; GG vs. GA + AA: *t* = 2.70, *P* = 0.017), but not in Asian populations (G vs. A: *t* = 1.71, *P* = 0.130; GG vs. GA + AA: *t* = 1.25, *P* = 0.250) (Figure 3).

## Discussion

This article investigated the relationship between EGF +61A/G polymorphism and HCC susceptibility. A total of 16 studies from 13 articles (2475 cases and 5381 controls) were finally included in this meta-analysis. Overall, the EGF +61A/G polymorphism was significantly associated with an increased HCC risk under all genetic models. However, considerable heterogeneity was detected across studies. Meta-regression showed that both ethnicity and study quality significantly contributed to the heterogeneity for EGF +61A/G polymorphism. Nevertheless, in the subgroup analyses by ethnicity and study quality, this significant association still existed in each subgroup, and the between-study heterogeneity became insignificant in Asian, European or African populations. Moreover, sensitivity analysis further strengthened the validity of the positive association in overall populations, and in Asian populations, indicating robustness of our results.

It is possible that the effects of genetic factors related to cancer are different across various ethnic populations. In this study, ethnicity was identified as a potential source of between-study heterogeneity by meta-regression and subgroup analyses. Although the reason for these discrepancies was not well known, some possibilities should be considered. First, there were significant differences in terms of +61G allele frequency among the three major ethnicities. The frequency of EGF +61G allele was greatest in Asian populations (65%), intermediate in European populations (42%), and lowest in African populations (30%). The higher HCC prevalence among Asian populations may be partly ascribed to the higher prevalence of EGF +61G allele. The frequency of EGF +61G among the controls of all studies was consistent with that in 1000 Genome Project, except for two studies [13,28]. The omission of these two studies did not substantially alter the results, indicating reliability of our results. Second, different linkage disequilibrium patterns may contribute to the discrepancy. The EGF +61A/G polymorphism may be in close linkage with nearby causal variant in one ethnic population, but not in another. Third, clinical heterogeneity such as age, gender ratio, life style and disease severity may also explain the discrepancy. The discrepancy might be due to genetic background and environmental exposure differences. Last but not least, owing to the limited number of studies in European and African populations included in this meta-analysis, the ethnic discrepancy was likely to be caused by chance. Therefore, further studies were needed to investigate the reason for this discrepancy.

Study design is an area of concern and can influence the interpretation of the results of meta-analysis. Among the eligible studies, there were 15 hospital-based studies, but only 5 population-based studies. Our results showed that EGF +61A/G polymorphism was significantly associated with HCC risk in hospital-based studies, but not in population-based studies. Therefore, the results should be treated with caution, because controls from hospital-based studies may not represent the general population. Larger population-based studies were required to further confirm the association between EGF +61A/G polymorphism and HCC susceptibility. Furthermore, according to chronic liver disease status in Asian controls, a significant association between EGF +61A/G polymorphism and HCC risk was obtained both in controls with chronic liver diseases, and in healthy controls, indicating reliability of the pooled results in Asian populations. Besides, all Asian studies were based on Chinese populations except for one Japanese study [14]. The frequency of EGF +61G allele was a little lower in Chinese populations than that in Japanese populations, according to 1000 Genome Project and our results. Geographical discrepancy should be considered in the analyses. The pooled results of these Chinese studies were consistent with those from Asian studies. Therefore, EGF +61A/G polymorphism may be associated with HCC risk in Asian populations, especially in Chinese populations. In addition, study quality was also identified as a potential source of heterogeneity by meta-regression. In this meta-analysis, 9 of the 16 studies were classified as high quality. Studies with low-quality design usually did not exclude those possible factors that may bias the estimate of the real effects and may result in incorrect conclusions. However, the association between EGF +61A/G polymorphism and HCC risk was significant in both high-quality and low-quality studies, suggesting that this bias cannot affect the final results.

Epidermal growth factor is a mitogen for hepatocytes, and plays a critical role in liver tissue regeneration, malignant transformation, tumor growth and progression [34]. Transgenic mice with liver-targeted overexpression of the secreted EGF fusion protein develop hepatocellular carcinoma, and blockade of EGF receptor activity halt the development and progression of HCC [35-37]. Thus, overexpression of EGF might be an important step toward development of liver cancer. For EGF +61A/G polymorphism, several studies have demonstrated that GG or GA genotype was associated with significantly higher EGF production both in normal peripheral blood mononuclear cell cultures and in serum and liver tissues of individuals [11,12,29]. It was thought that EGF +61A/G polymorphism might be correlated to HCC. Our results showed that EGF +61G allele was significantly associated with an increased HCC risk, which was consistent with the hypothesis. However, the molecular mechanism of the association between EGF +61A/G polymorphism and HCC risk remains relatively unclear.

To our knowledge, this present meta-analysis is the most comprehensive one related to the relationship between EGF +61A/G polymorphism and HCC risk. Compared with the previous meta-analysis [38], another eight studies were included in this meta-analysis. The sample size of total participants in our study (2475 cases and 5381 controls) was much larger than that in the previous one (1304 cases and 2613 controls). Thus, the pooled results were more reliable and robust in our study. Furthermore, the quality of the included studies was evaluated in our study, but not in the previous one. Meta-regression was performed to explore the sources of heterogeneity among studies, which allowed a more thorough examination and appropriate qualification of our results.

Despite our efforts in performing a comprehensive analysis, several limitations should be considered. Firstly, obvious publication bias was detected in overall populations. Bias may result from our exclusion of unpublished data, as well as studies published in languages other than English and Chinese. Secondly, the controls were not uniformly defined. Some studies were population-based, while others were hospital-based. Considering the overwhelming impact of chronic liver diseases on HCC development, controls were divided into healthy controls and controls with chronic liver diseases. The subgroup analyses showed that the significant association between EGF +61A/G and HCC was present both in healthy controls and in patients with chronic liver diseases, indicating the role of EGF +61A/G in the risk of HCC, regardless of type of controls. Moreover, the pooled ORs for individuals with chronic liver diseases were higher than those for healthy controls under all genetic models. Therefore, the chronic liver diseases may change the environment in vivo and mediate the ability of genetic factors to contribute to HCC. More studies should be designed to investigate the role of EGF polymorphisms in combination with chronic liver diseases in HCC pathogenesis. Thirdly, our meta-analysis was based on unadjusted estimates. If individual data were available, adjusted estimates by confounding factors could be obtained to conduct a more precise analysis. Fourthly, gene-gene and gene-environment interactions were not addressed in our meta-analysis due to lack of sufficient data. Aside from genetic factors, other factors such as exposure to aflatoxin B1, high cigarette smoking, and habitual alcohol abuse might also play vital roles in the development of HCC. However, we could not perform subgroup analyses based on environmental exposure owing to the limited reported information on such associations in those included studies. Finally, the number of studies included in the meta-analysis for European populations and African populations was relatively small, which may lead to low statistical power and generate fluctuation in estimation.

## Conclusions

In summary, this meta-analysis suggests that EGF gene +61A/G polymorphism is significantly associated with the increased risk of HCC, particularly in Asian populations. Further well-designed and large-scale studies with the consideration of gene-gene and gene-environment interactions should be conducted to investigate the association in different ethnic populations.

## Additional files

Additional file 1: Table S1. Scale for Quality Assessment.
Additional file 2: **Checklist S1.** PRISMA checklist.

## Abbreviations

EGF: Epidermal growth factor
HCC: Hepatocellular carcinoma
CBM: Chinese BioMedical Literature
CNKI: Chinese National Knowledge Infrastructure
OR: Odds ratio
95% CI: 95% confidence interval
HBV: Hepatitis B virus
HCV: Hepatitis C virus
SNP: Single nucleotide polymorphism
5′-UTR: 5′-untranslated region
PRISMA: Preferred Reporting Items for Systematic Reviews and Meta-analyses
HWE: Hardy-Weinberg equilibrium
PCR-RFLP: Polymerase chain reaction-restriction fragment length polymorphism assay
