# Supplementary material for: Association between epidermal growth factor gene +61A/G polymorphism and the risk of hepatocellular carcinoma: a meta-analysis based on 16 studies
Source: BMC Cancer. 2015 Apr 25;15:314. doi: 10.1186/s12885-015-1318-6 (PMC4418097; doi:10.1186/s12885-015-1318-6)
Supplement: Additional file 1: Table S1. — Scale for Quality Assessment. [file 12885_2015_1318_MOESM1_ESM.docx]

| Additional file 1: Table S1. Scale for Quality Assessment | |
| --- | --- |
| Criteria | Score |
| Source of cases |  |
| Selected from population or cancer registry | 3 |
| Selected from hospital | 2 |
| Selected from pathology archives, but without description | 1 |
| Not described | 0 |
| Source of controls |  |
| Population-based | 3 |
| Population-based and Hospital-based mixed | 2 |
| Hospital-based (cancer-free patients) | 1 |
| Not described | 0 |
| Ascertainment of cancer |  |
| Histopathologic confirmation | 3 |
| Histopathologic confirmation and Patient medical record mixed | 2 |
| Patient medical record | 1 |
| Not description | 0 |
| Case-control match |  |
| Matched by age and gender | 3 |
| Matched only by age or by gender | 1.5 |
| Not matched or not described | 0 |
| Specimens used for determining genotypes |  |
| White blood cells or normal tissues | 3 |
| Tumor tissues or exfoliated cells of tissue | 0 |
| Quality control of genotyping |  |
| Different genotyping assays confirmed the result | 3 |
| Quality control by repeated assay | 1.5 |
| Not description | 0 |
| Hardy-Weinberg equilibrium in controls |  |
| Hardy-Weinberg equilibrium | 3 |
| Hardy-Weinberg disequilibrium | 0 |
| Total sample size |  |
| >1000 | 3 |
| >500 and <1000 | 2 |
| >200 and <500 | 1 |
| <200 | 0 |
